# The Lived Experience of Mothers of Children Who Have Been Sexually Abused‐ an Interpretative Phenomenological Analysis

**DOI:** 10.1002/jclp.70057

**Published:** 2025-10-21

**Authors:** Dympna Browne, Donncha Hanna, Julie‐Ann Jordan, Clare Howie, Kevin F. W. Dyer

**Affiliations:** ^1^ Psychological Services Belfast Health and Social Care Trust Northern Ireland; ^2^ School of Psychology Queen's University Belfast Northern Ireland; ^3^ Child Care Centre Belfast Health and Social Care Trust Northern Ireland; ^4^ IMPACT Research Centre Northern Health and Social Care Trust Northern Ireland; ^5^ Psychological Therapies Service Northern Health and Social Care Trust Northern Ireland

**Keywords:** Child sexual abuse, interpretative phenomenological analysis, non‐offending mothers, shame, trauma

## Abstract

**Objective:**

Mothers of children who are survivors of sexual violence experience significant distress from the discovery of their child's abuse. Previous research has mostly focused on the mental health symptoms arising from this type of trauma, rather than the personal journey and meaning mothers take from these incidents. This study aimed to examine the lived experiences of non‐offending mothers of children who have been sexually abused.

**Methods:**

Six mothers, aged 34–53 years‐old, whose children had experienced sexual abuse took part in the study. All participants were attending a specialist multidisciplinary service in Northern Ireland for the investigation and treatment of sexual violence. Semi‐structured interviews were conducted and content was analysed via interpretative phenomenological analysis.

**Results:**

Three superordinate themes emerged from the interviews: (1) “The Sins of the Mother”, comprising three subordinate themes centering on negative evaluations of the self and perceived judgements from others; (2) “Impotent Anger”, relating to injustice and revenge fantasies; and (3) “It Changed Me as a Parent”, comprising two subordinate themes on how the mothers perceived and parented their child after the abuse.

**Conclusions:**

Mothers perceived the abuse of their child as incompatible with their view of themselves as good parents, leading to internalized feelings of shame as well as externalized feelings of anger and injustice. This had implications for the mothers' relationship with their child and confidence in parenting.

Non‐offending parents of children who experienced sexual abuse have been described in the literature as ‘secondary victims’ as they face significant emotional difficulties following the traumatic event (Cyr et al. 2016). In addition to the negative impact on their own psychological health, such sequelae can affect their ability to support their child during this critical time (Elliott and Carnes 2001; Knott and Fabre 2014). While many parental figures (e.g., biological fathers, adoptive parents) can fall within this primary caregiver group, the majority are biological mothers who have had no involvement in the sexual offence (Malloy et al. 2005). For these ‘non‐offending mothers’, learning that their child has been abused marks the beginning of a series of life‐changing challenges and potentially aversive experiences.

How mothers come to the realization of their child's abuse varies greatly (McElvaney 2015). Some children unintentionally reveal possible abuse through behaviours or unplanned statements, while others make tentative disclosures to test responses before either retracting or proceeding with the disclosure. The least common disclosure pattern involves children making direct and purposeful disclosures after an initial abusive act (Alaggia 2004; Sorensen and Snow 1991). In an exploratory study of the disclosure process with 125 mothers, it was found that, although the majority of participants were unaware of the abuse before disclosure, over half were suspicious that “something wasn't quite right” (Plummer 2006, p.1232).

In 2021, the UK Home Office published their ‘Tackling Child Abuse’ strategy (Home Office 2021), highlighting the need for therapeutic support for non‐offending parents. While the professional literature no longer promotes the notion of maternal blame and collusion, there is some evidence that such beliefs persist in child protection services (Leonard 2013; Scott 2022) and in general society (Davies et al. 2013; Zagrodney and Cummings 2020). Qualitative studies comment that the system can be unsupportive and potentially traumatizing (Plummer and Eastin 2007; Kilroy et al. 2014; McElvaney and Nixon 2020). Mothers have described feeling undermined by healthcare professionals and that their emotional responses to allegations are excessively scrutinized with negative judgements made about their mental stability and parenting capacity (Plummer and Eastin 2007). Paradoxically, non‐offending mothers not only feel unfairly blamed by others for the abuse, but they also frequently blame themselves (Kilroy et al. 2014). Hooper (1992) reported that they experienced an ‘unspecific guilt’ about failing to protect their child. A myriad of potential reasons may lie behind such attributions of self‐blame, including the mothers' relationship with the perpetrator; failing to notice signs of the abuse; and attending to their own feelings of distress instead of focusing solely on the child (Kilroy et al. 2014; McCallum 2001; Plummer and Eastin 2007).

Perceptions of self‐blame and negative evaluation by others in combination with the fundamental trauma of their child's abuse can, understandably, provoke significant psychological distress in this population. Evidence suggests that parents are at heightened risk of developing PTSD and clinical depression following their child's disclosure (Cyr et al. 2016; Daignault et al. 2021; Knott and Fabre 2014). However, a number of factors can moderate the impact of these experiences on wellbeing. Levels of child PTSD symptomology have been found to be concordant with the severity of traumatic stress reactions in non‐offending parents (Mangold et al. 2022). General factors such as child age, child gender, severity of abuse, and environmental factors may also play a role, but the extant evidence is conflicting (Elliott and Carnes 2001). More substantive psychological and relational processes have received stronger support as possible correlates. Avoidant coping strategies, intrafamilial abuse, and insecure parent‐child attachment have emerged as related to psychological distress (Bolen and Lamb 2004). These can be exacerbated by mandatory legal and safeguarding responsibilities of non‐offending mothers (e.g., testifying in court), which further compounds their susceptibility to poor mental health (Burgess et al. 1990).

To date, most of the limited research on non‐offending mothers of children who experienced sexual abuse has been aimed at understanding their parenting responses to their child or the impact of the trauma on their mental health. The aim of the present study is to go beyond labelling the symptomatological consequences and instead focus on the lived experience of the mothers. The existing research literature implies this is a unique, vulnerable, and overlooked population with limited understanding of how non‐offending mothers make sense of the abuse‐related events and their sequelae. Such information will likely foster development of bespoke formulation and intervention approaches, which are lacking in this area. A qualitative approach was adopted in this investigation using Interpretative Phenomenological Analysis (IPA; Smith et al. 2009) to provide insight into these clinically‐relevant issues. Whilst IPA has become increasingly high profile in health and clinical psychology, it has yet to be utilised as a research methodology with parents of children who have been abused. IPA is uniquely suited to examining this population considering the following: (a) the area is complex, ambiguous and emotionally laden requiring exploration of the nuance of mothers' psychological experience (Smith and Osborn 2015); and (b) the population is niche with an in‐depth interpretive analysis of a smaller sample more appropriate for understanding the unique mechanisms at play. By exploring the individual experiences and perceptions of non‐offending mothers using this methodology, the study sought to increase current knowledge of key psychological processes relevant to this major life event and to supporting this at‐risk population.

## 1 Methodology

### 1.1 Participants

Participants were recruited from a specialist multidisciplinary service for the investigation and treatment of sexual abuse in Northern Ireland. Purposive sampling was used and potential participants were contacted via referrals from therapists in the service. In total, seven mothers were contacted and six agreed to participate. All participants were aged between 34 and 53 years old. Their children were aged between 5 years and 15 years old, with five out of the six children being female. The average duration of the abuse was approximately 2 years although most participants were unclear about the onset given the secretive nature of abuse and the young ages of the children. The length of time since discovery ranged from 18 months to 5 years. Three of the mothers were single parents and all perpetrators were male.

### 1.2 Procedure

All procedures were fully compliant with Northern Ireland laws and health and social care service policies. Ethical approval was obtained from Office of Research Ethics Committees Northern Ireland (ORECNI; 29/04/2014, REC Ref: 14/NI/0039). Therapists provided mothers with a Participant Information Sheet. Individuals interested in taking part were contacted by the research team and provided with further details about the study. If the mother agreed to participate, an interview was arranged at a location of their choice (e.g. home, Child Care Centre) and informed written consent was obtained. A semi structured interview schedule (see Appendix 1) was used as a framework for discussion. The interview schedule was developed by a consultant clinical psychologist working in the area of child sexual abuse, and reviewed by clinical social workers and a family therapist who also worked in child sexual abuse. In line with IPA guidance, the interview schedule was short and open ended, supplemented with prompts, thereby exploring important areas of the literature while also allowing flexibility in answers and in depth expression of lived experience. Interviews lasted between 45 and 90 min, and were recorded and transcribed verbatim. The interviews were conducted by the lead author who at the time had worked as a consultant clinical psychologist, specialising in both the investigation and treatment of child sexual abuse for the past 5 years and had completed extensive training in qualitative methods.

### 1.3 Analysis

The study employed a qualitative design and adhered to the IPA guidelines of Smith et al. (2009). Specifically, each transcript was read and reread by the first researcher to engage with the transcribed information, before moving on to “initial noting” to highlight phenomenological descriptions and make interpretative comments. Coding data into emergent psychological themes involved categorising comments from each transcript and interpreting them in light of both the participants' narrative and the experience of the researcher. The lead author engaged in researcher reflexivity through self‐ reflection and seeking feedback from the research team to ensure examination of assumptions. A visual mapping exercise of emergent themes allowed thematic cross‐connections to be explored. Three researchers (D.B., D.H., & K.D) conducted credibility checks to provide deeper insight and analysis into the phenomenological experiences of the participants, as well as to counter any bias and assumptions in the lead author's analysis. The emergent themes were then organised into superordinate and subordinate themes. The interpretation offered in the analysis is acknowledged to be only one of many possibilities and generalizable claims are made cautiously in the context of the previous findings. Mother pseudonyms have been used in quotes to preserve anonymity.

## 2 Results

Analysis highlighted the following three inter‐ related themes as prevalent within the data: ‘The Sins of the Mother’; ‘Impotent Anger’ and ‘It Changed me as a Parent’ (Table 1).

**Table 1:** Superordinate and Subordinate Themes.

Superordinate theme	Subordinate theme
The Sins of the Mother	As a mother, I should have known.
	How others view me now.
	The self in relation to others.
Impotent Anger	The quest for justice.
	He's still out there.
	Revenge fantasies.
It Changed me as a Parent	It changed how I see my child.
	It changed how I parent my child.

### 2.1 Sins of the Mother

The first superordinate theme captures the participant's sense of themselves as ‘bad’. They describe feeling that the abuse of their child is tangible evidence, to both themselves and to others, of their deep rooted inadequacies and incompetence. This is summarised by Louise who says ‘*the shame is the feeling that I brought this into her life’*. Feeling that in some way they caused the abuse to happen was intensely painful for these mothers. The hurt associated with this belief seemed to intensify in the presence of others with the expectation of negative evaluations by those individuals. Loss of trust in the mothers' own capacity to protect alongside a belief that others are unsafe and untrustworthy, contributed to a reluctance to access social support. The Sins of the Mother is made up of three subordinate themes: ‘As a mother I should have known’, ‘How others view me’ and ‘The self in relation to others’.

#### 2.1.1 As a Mother I Should Have Known

A version of ‘as a mother I should have known’ was mentioned by each of the participants. This indicates an idealized view of mothers as all‐knowing and intuitively protective. The fact that they had failed to live up to this standard led them to feel partly culpable for the abuse. All of the participants had a sense of self‐blame that impacted negatively on their identity as a mother.

I should have known. Like I knew there was something with Jane, I always knew there was something but I didn't know what. I didn't think it was that at all like. I really didn't think it was that. It just made me feel bad as a mother not to have noticed that. To have had that in my own house and not to have known it was going on.

(Cara)

The experience of feeling ‘bad’ as a mother was common to all of the participants. Most spoke about how with hindsight they could see how they misattributed clues of the abuse to other causes. Louise describes her experience of her daughter refusing to go to her father for weekend visits. She recognised that her ‘past self’ did not know about the abuse and therefore rationalised her daughter's behaviour. However, her ‘present self’ perceived that she was ‘forcing it’ and therefore enabling the abuse to occur.

At the time I made sense of it as in you know it's a stage she's going through. So it's hard knowing I was forcing it. I was kinda always making sense of it at the time when I should have been saying that there's something wrong here.

(Louise)

Maria stated adamantly that nobody can do or say anything to take away her guilt, suggesting she feels beyond redemption and her self‐blame is now permanently embedded in her identity.

The guilt will always be with me, nothing anyone can do or say will take away that. I mean I'm sure I have done things that I'm not proud of and that weren't nice you know and I think to myself, Sarah's being punished for the sins of the mother.

(Maria)

#### 2.1.2 How Others View Me Now

Louise describes how speaking to others further compounded feelings of self‐blame.

He was the first person that I'd spoken to and then I kind of became aware of shame, here's me now talking about this thing and it's kind of hard to articulate but it's like this wave of shame then about this you know, the awfulness of it or whatever.

(Louise)

Each of the participants in their own way described feeling blamed by and disapproved of by significant others such as family, friends, and involved professionals. While sometimes participants reported being treated with empathy, at other times they were greeted with disbelief. Maria describes managing the discomfort associated with the judgements of others by rejecting the importance of relationships with others and, instead, focusing on her relationship with her daughter.

I'm sure people are saying how did she not know because to be honest before this I would have probably been one of them people. I'll always remember seeing things on tv and stuff and saying or reading stories in the magazines and saying how can a mother not know. It doesn't matter anyway the only thing that matters is Sarah.

(Maria)

The majority of the participants commented on the stigma of sexual abuse and expressed concern about keeping it private to protect their child's identity. As reflected in Annie's words, many felt that the only way to escape potential exposure was to relocate or withdraw from relationships.

We need to get out of that area because I'm worried about it coming out in the newspapers too and people are going to figure out that it's my wee boy. … I'm not ashamed of him, I just don't want it to affect the rest of his life.

(Annie)

#### 2.1.3 The Self in Relation to Others

Throughout the interviews a recurring theme was how participants' views of ‘others’ and ‘themselves in relation to others’ had altered. Annie describes her disconnection with others and inability to have intimate relationships since the disclosure.

It's just affected my whole life, the way I think about people, and the way I judge people…I had a partner at the time but me and my partner split up because I can't get close to anyone, I can't be with anyone, my priority is to look after my kids, I just explained that to him.

(Annie)

Janice repeatedly mentions her ‘major trust issues’. For Janice, the discovery that a man she had known for 15 years had abused her children leads her to question her judgement of others and her decision making in relation to others. The fact that even those closest to her are now under scrutiny means that her trust issues are all‐encompassing and the world truly is a dangerous place.

I have doubts. I know I can trust my husband and my own parents, But with everything that happened‐ there is just this wee niggle in the back of your mind – what if – what if. You believed you know that man and look what happened.

(Janice)

Similar to other participants Janice has a newfound sense of responsibility towards other children. However for her, this sense of responsibility is conflicted with complex feelings including her fear regarding how others might also see her as a potential perpetrator.

Even when I'm out with my own children and say you're in a shopping centre and a wee child gets lost, my first instinct is to go and get them because I'm afraid of somebody else coming along and taking them and doing things with them. But again, I have to be so careful because I'm afraid of someone thinking I'm going to harm them.

(Janice)

### 2.2 Impotent Anger

Without exception, each of the participants conveyed intense feelings of anger since the abuse of their child was revealed to them. From their descriptions it was evident that they felt powerless to act constructively on the anger. Without the ability to release this pent up emotion, this anger did not dissipate over time but instead, anger became part of participants' daily experience. Impotent Anger as a superordinate theme is made up of the following subordinate themes; ‘The quest for justice’, ‘He's still out there’ and ‘Revenge fantasies’.

#### 2.2.1 The Quest for Justice

Five out of the six participants reported the judicial system as being inaccessible and inadequate for children. The participants' anger seemed to be associated with feeling unsupported and discounted in a system in which they had hoped would provide justice. Annie highlights the sense of unfairness experienced by the participants who perceived the court as supporting the perpetrator rather than the victim, causing her to feel sidelined and invisible.

When I went to court last week, it was all about him. He needs a report done. We need to test his IQ because he's refusing to speak to anybody. Him and his family need this support. Not once did we get mentioned. What support are you going to give me and my family?

(Annie)

Some participants experienced the dilemma of having to decide whether to proceed with court. This decision involved weighing up whether to allow their child to be a witness to the court and therefore subjecting them to the adversarial nature of the proceedings with no guarantee that it would have a positive outcome.

Everything is about protecting them. It's not about protecting the children. Like if my daughter had gone to court, although they would have said that they would have talked nice to her, they would have ripped her to shreds and made her out to be a liar.

(Janice)

The majority of the participants described feeling a sense of futility about their quest for justice. For Cara, a sense of closure seems to be about being believed by others more so than seeking vengeance.

It went on for 2 years, but it ended up anyway that there wasn't enough evidence to bring it to Court so you're still in that limbo. Nothing's being done, well she feels nothing's been done about it, that he just walked away from it. I think that if he had been convicted, there would have been closure. It would have been (for child), they believed me rather than nobody believed me.

(Cara)

#### 2.2.2 He's Still out There

In the absence of a conviction, participants described feeling that they were burdened with the responsibility for the safety of other children.

Now you have this extra worry about him attacking other children.

(Annie)

Donna describes anger directed at the perceived freedom and invisibility of the perpetrator which was contrasted to their own feelings of being trapped and exposed.

That thing, wherever he is now, has sort of like wrecked our lives. And he's probably now on the streets, having, continuing with what he's doing. Cause I believe he's out. While we are all the rest of us are just suffering because of his actions.

(Donna)

For Louise, even ordinary events do not provide her with an escape or respite from this feeling of anger, rather in fact it is ordinary events being interrupted that elicit this anger. Louise's anger seems to be linked to her struggle in making meaning of this experience as she is unable to exert influence and power over what she believes to be a dangerous situation for other people's children.

I know I'm angry and that feels wrong and it saddens me because before this I never had that feeling. It's not as if I've never been angry in my life but it's the nature of the anger I feel. I recently, I was going about my business and I saw his car and it was right beside a children's play area and I just felt so cross and then I was cross because what do I do?

(Louise)

#### 2.2.3 Revenge Fantasies

Each of the participants spoke of an overwhelming urge to act out violently towards the perpetrator, particularly immediately after the disclosure.

I wanted to get a knife and stab him to death.

(Maria)

Many participants described how over time, with the dynamics of perceived injustice and a concern about the perpetrator's whereabouts, they struggled with ongoing revenge fantasies. Janice describes her fear that she will be unable to control her anger if she comes face to face with the perpetrator.

If I'm out somewhere and I see his face I would not be responsible. I wouldn't be able to control myself; honestly I wouldn't be able to stop myself (Janice starts crying). Things like that frighten me because I can't control it. It's the one part of my life that I can't control. Everything else like trying to keep them safe there is a control element in it but that I can't control.

(Janice)

Donna was the only participant who had actually acted out violently towards the perpetrator. She describes the power she felt while she attacked the perpetrator illuminating how for all participants the revenge fantasies served to reassert their role as a protective parent.

The best feeling ever. He took something from her so I was taking something from him. That's the way I felt and I don't regret it. Even to this day I don't regret it. I'd do it again.

(Donna)

It should be noted that Donna's anger is in the context of her own history of childhood sexual abuse, and the activation of distressing memories which she said were ‘well hidden’ before her daughter's disclosure. It appears that in that moment Donna was disoriented and unable to discriminate her own abuse from her daughter's abuse.

I think when I was beating him I was also beating the ones that done it to me.

(Donna)

For others with no history of being personally abused, their revenge fantasies caused psychological distress and disturbed their previously held assumptions about themselves. As Janice said:

I've never wanted to hurt anybody in my whole life and a lot of my life has been about looking after others or helping in different ways.

(Janice)

### 2.3 It Changed Me As a Parent

A dominant concern for all of the participants was their relationship with their child. They all described contemplating the negative impact of the abuse on their child. In doing so this seemed to trigger their feelings of self‐blame, which in turn affected their responses to their child. This superordinate theme is made up of two subordinate themes: ‘It changed how I see my child’ and ‘It changed how I respond to my child’

#### 2.3.1 It Changed How I See My Child

All but one of the participants described their child's identity as being irreparably damaged or marred in some way by the abuse. These participants reported feeling loss for the innocent child pre‐abuse and a struggle to fully accept their child post‐abuse. Maria previously made sense of having a baby despite being sterilized to indicate that her daughter was here for a ‘reason’. However, as a result of the abuse, the idealized narrative that Maria had around her daughter being extraordinary and special is now meaningless to her.

there's a reason she survived it through sterilisation you know. I mean I think to myself now ‐ why did she survive it, to be punished to be hurt…He's just wrecked her wee life.

(Maria)

For some participants, the abuse not only changed how they saw their child after the disclosure but also before the disclosure. Janice re‐interprets her child's past in light of her current knowledge of the abuse, and has embedded the impact of sexual abuse into her image of her daughter's past, present, and future identities.

At certain points in her life when she's been having trouble you can pinpoint her behaviour as a response to the sexual abuse and as time has gone past you can relate it to more of the person she is and who she is growing into.

(Janice)

Louise is the only participant who did not report believing that her daughter was in some way damaged forever because of the abuse. She attributes this to her daughter's young age, which protects her from ‘*carrying a sense of shame’*.

One of the things that I've learnt, that is a saving grace, is that my wee one is young and if she had been older then maybe she'd be carrying a sense of shame. But as far as she is concerned, yes it happened to part of her but it could have been her knee so I just think if that's what I find myself carrying for a period of time that's ok, I know its not going to be forever.

(Louise)

#### 2.3.2 It Changed How I Respond to My Child

It was with a sense of sadness and hopelessness that each of the participants described a loss of confidence in their parenting following the abuse discovery. Participants were no longer secure in their parenting role and were particularly vulnerable to feeling this anxiety when faced with typical parenting stressors. Cara now attributes her daughter's negative behaviour as being a consequence of the abuse. Cara attempts to ‘make up for it’ by giving into the child's demands, however this has undermined her authority as a parent.

You try to make up for it. Like I wouldn't have been as short with her as I was with my older daughter and son. …. So then I would be more lenient on her but it kinda backfired on myself now. … you need boundaries and she doesn't like boundaries now.

(Cara)

Participants demonstrated a strong desire to protect their child from negative emotions as well as potential physical danger. For many, social isolation was the only way of ensuring their child's safety. Janice perceives herself as contributing to her children's loss of a ‘normal childhood’ as a result of her own fearful responses.

When you're a child, you're supposed to be a child, having fun, having adventures, learning new things making new things, that's all been taken away. Because not only are my children more cautious of what to do but for me as a parent I'm terrified, I'm literally terrified. My children don't play on the street, they play in the back garden and even if I hear voices talking over the fence I'll bring them into the house because I don't want anyone near them.

(Janice)

In contrast to other participants who feared for their child's future and mourned the loss of an expected relationship with their child, Louise describes being proactive in determining the course of their relationship and in imagining her child's future.

Cause at one stage I thought I could easily turn into a campaigner and it could dominate my life ……But the things that are important is I think when my wee one looks back to when she was 5 years old and she has an experience of her mummy being really silly or going to the beach or doing really ordinary things and that's hugely important.

(Louise)

## 3 Discussion

This study highlighted important issues in relation to the identity and perceived maternal worthiness of mothers whose children have been sexually abused. The first theme “Sins of the Mother' focused on participants' experience of self‐blame and self‐denigration following discovery of the abuse. The second theme ‘Impotent Anger’ illustrates how the mothers were left with anger at the injustice of the crime but were powerless in expressing this complex emotion. The final theme ‘It Changed me as a Parent’ revealed insight into perceived relationship changes fomented by the abuse and its legacy. Mothers felt insecure and isolated as parents, with concerns that a victim identity had been imposed upon their child that may persist across a lifespan.

The finding that mothers blamed themselves for the abuse of their child is supported by an existing body of studies (Hooper 1992; Kilroy et al. 2014; McCallum 2001; Plummer and Eastin 2007). However, the present study illustrates the subtle and insidious way self‐blame attributions can impact on mothers' sense of self image and relationships with others. Their sense of self‐blame was experienced as all encompassing. Not only did they describe feeling bad about themselves as mothers but they also alluded to negative feelings about all facets of themselves. Self‐blame is a common reaction to traumatic events as it promotes a sense of coherence and predictability in a ‘just world’ where people get what they deserve (Lerner 1980). Trauma theorists have proposed that there are two types of self‐blame: behavioural and characterological, which differ substantially in terms of their implications for a person's identity (Janoff‐Bulman 1979). Behavioural self‐blame is associated with specific actions or omissions by a person. Characterological self‐blame on the other hand is associated with attributions about the self as unworthy. The participants in this study describe experiencing aspects of both types of self‐blame. For example, Janice's narrative was at times reflective of behavioural self‐blame as she recalled how she should have changed her response to her child. On the other hand, Maria highlighted her ‘sins’ as a causative factor in her child's abuse. This appears to be more consistent with characterological self‐blame as she focused more ‘on the past and a question of deservedness rather than avoidability’ (Janoff‐Bulman 1985).

Theories of self‐conscious emotion suggest that individuals who make internal, stable, and uncontrollable attributions associated with characterological self‐blame are prone to shame rather than guilt (Tangney et al. 2007). The findings from the present study imply that shame reactions may constitute a prominent overarching emotional abstraction that reflects the lived experience of mothers whose children have been sexually abused. To manage overwhelming feelings of shame, Nathanson and American Psychiatric Association (1987) argued that individuals employ four coping styles: withdrawal from others, avoidance of others, attacking the self, and attacking others. All participants in this study described not only feeling they had fallen short of the standards of a ‘good mother’, but actually saw themselves as the embodiment of a ‘bad mother’. This type of an internal appraisal could represent a manifestation of the “attack self” shame coping response. In contrast, the ‘Impotent Anger’ subtheme of ‘Revenge Fantasies’ may reflect the “attack other” coping style and a desire to expunge shame feelings by externalizing anger onto others. Considering the presence of a clear target for such feelings in the form of the perpetrator, outward anger and urges for retribution could deflect shame arising from mothers' perceived inability to protect their child (Nathanson 1992). Tangney and Dearing (2002. p23) describe anger as ‘reactivating and bolstering the self’. It may be that mothers' extreme revenge fantasies represent a coping mechanism to counter a sense of powerlessness associated with the injustice of their situation and their ruptured parental role, serving a purpose of reasserting themselves as a protector. However, for some of the participants the violent nature of their fantasies were shameful in themselves as it conflicted with their self‐image before the abuse (Scheff 2014).

Participants reported significant difficulties within relationships, which is often one of the primary ‘reporting costs’ of sexual abuse for mothers (Massat and Lundy 1998). Throughout all of the accounts, feelings of inadequacy were felt most acutely when in the presence of others, resulting in feeling diminished and exposed. As they felt unable to judge the trustworthiness of others, this seemed to affect their ability to feel secure enough to show vulnerable aspects of themselves in relationships. Shame is understood as an emotion that is activated when in the presence of real or perceived negative evaluation from others, often resulting in utilizing “avoidance” or “withdrawal” coping styles (Nathanson 1992). It follows that mothers of abused children may therefore experience other people as sources of anxiety and intimidation, even those who are close to them.

The impact of childhood sexual abuse on parenting emerged as a key theme across the interviews. This is supportive of a significant literature base highlighting the challenges of parenting following discovery of abuse (Elliott and Carnes 2001). Participants appeared to hold a different image of their child following the abuse. This is in line with research indicating that the sexual abuse label may lead to lowered expectations and skewed negative beliefs in parents (Kouyoumdjian et al. 2009). Many of the mothers described a feeling of loss, perceiving that the abuse had effectively robbed them of their child as they had known them and replaced them with a child who was now irreversibly damaged (Hooper 1992). This is likely to reflect exaggerated cognitive biases that have become entangled with their own feelings of self‐blame about contributing towards the abuse – a retrospective image of their child as perfect and themselves as a protective parent is replaced with the image of their child as traumatised and they as an inadequate mother. It is perhaps unsurprising in these circumstances that participants reported difficulty with setting limits and discipline in the ‘It changed how I respond to my child’ subtheme (Plummer and Eastin 2007). Heightened emotion in a child could activate the parent's sense of self‐blame about the abuse and provoke a parenting response of acquiescing to demands, reassuring the child of their love or by withdrawing from the interaction. Whilst this range of responses seemed to initially reduce the pain felt by the parents about their feeling of self‐blame, accounts from parents whose children were abused in the more distant past indicated that overtime this had a detrimental affect on the relationship between the parent and the child.

### 3.1 Clinical Implications

The present study provides a unique insight into the lived experience of mothers of children who have been sexually abused and highlights areas of concern for clinicians working with this population. Mothers reported an undermined sense of self, with the abuse experience provoking powerful self‐perceptions of being a defective parent that impacted negatively on their relationship with their child. Models of posttraumatic shame in conjunction with some of the thematic material highlighted in the current investigation (e.g., “impotent anger” tendencies, maternal perception of child victim identity) have relevance as predisposing and perpetuating factors in clinical formulations for this population. Incorporation of these concepts within existing approaches (e.g., biopsychosocial formulation model; Macneil et al. 2012) may be helpful in both individual and family therapy (see Gilbert 2010; Nathanson 1992). Moreover, considering mothers reported that post‐abuse experiences often contributed to the development of less effective parenting styles (e.g., permissive, overprotective), treatment plans involving psychoeducation and parenting strategies would be useful in avoiding such outcomes. For example, distorted views of probable risk combined with a lack of confidence in ability to discern safe from unsafe people – as reported in the present study – has been shown to be associated with overprotective behaviours in parents (Davies 1995), which can lead to further posttraumatic stress and anxiety in children (Rowland‐Klein and Dunlop 1998). Considering the primary emotional challenges facing mothers and their children who have experienced abuse, it is incumbent upon clinicians to provide guidance on common reactions and corrective interventions to prevent development of secondary difficulties post‐abuse (e.g., misplaced parental self‐blame, child conduct difficulties).

### 3.2 Limitations

Although all the participants in this study shared common themes, they were a heterogeneous sample group. The heterogeneity of the sample in terms of the quantity of therapeutic sessions is a limitation of this study as the acquisition of post‐trauma coping skills is a developmental process (Reynolds et al. 2017). Further IPA research purposefully selecting specific pertinent factors to include in samples such as intrafamilial and extrafamilial abuse experiences, as well as the ages of the children and the abuse experiences frequency and severity, would enhance applicability of the findings. A useful inclusion in further studies would also be to obtain self‐report baseline measures of symptoms (e.g., PTSD, depression) as further descriptors of sample characteristics; however, this would need to be balanced with the potential for participant burden.

The current focus on mothers rather than fathers, while useful in the current research, prevents understanding of the full parental experience of this population. The psychological reactions of fathers in this unique scenario may be distinctly different, and future research should examine their personal experiences. In a related issue, the themes in the present study also may not capture the views of parents who declined to participate, as their reluctance may indicate distinct psychological processes at play. Participants in this study were self‐selecting as they were all attending with their child for therapeutic support and this may have led to a sample with a skewed level of coping skills. Additional qualitative and large‐scale controlled quantitative studies in this area to fully explore the psychological factors relevant to this population would be beneficial.

## 4 Conclusions

Mothers of children who have been sexually abused reported a number of themes centring on self‐blame, an undermined sense of self, impotent anger, and perceptions of a deeply altered relationship with their child. Shame theories provide a useful framework of understanding of these issues, particularly relating to self‐directed and perpetrator‐directed anger. The emotional experience of mothers clearly impacted on their capacity to engage with others and their confidence in parenting. Services involved with this population should provide tailored supports (e.g., psychoeducation, parenting strategies) to avoid escalation of such difficulties.

## Supporting information

Appendix 1 interview schedule.

## Data Availability

The data that support the findings of this study are available on request from the corresponding author. The data are not publicly available due to privacy or ethical restrictions.
