# Supplementary material for: The Lived Experience of Mothers of Children Who Have Been Sexually Abused‐ an Interpretative Phenomenological Analysis
Source: J Clin Psychol. 2025 Oct 21;82(2):198–206. doi: 10.1002/jclp.70057 (PMC12793815; doi:10.1002/jclp.70057)
Supplement: Supplementary file 1 — Appendix 1 interview schedule. [file JCLP-82-198-s001.docx]

**Understanding the emotional experiences of parents.**

**Interview Schedule**

Can you tell me about what happened to your child?

(prompt: who was perpetrator, relationship with perpetrator, circumstances around disclosure, services involved since disclosure)

Can you tell me about how your child’s experience of sexual abuse has affected your life?

(Prompt: relationship with child, relationship with family, relationship with friends, relationship with friends, neighbours, work)
